# Rosuvastatin-Based Lipid-Lowering Therapy for the Control of LDL Cholesterol in Patients at High Vascular Risk

**DOI:** 10.3390/jcm13071894

**Published:** 2024-03-25

**Authors:** Jose María Mostaza, Carlos Escobar

**Affiliations:** 1 Lipid and Vascular Risk Unit, Department of Internal, University Hospital La Paz-Carlos III, 28046 Madrid, Spain; josemaria.mostaza@salud.madrid.org; 2 Cardiology Department, University Hospital La Paz-Carlos III, 28046 Madrid, Spain

**Keywords:** cholesterol, atherosclerotic vascular disease, rosuvastatin

## Abstract

Vascular diseases are the leading cause of death in Spain. Hypercholesterolemia is not only a cardiovascular risk factor, but also underlies the etiopathogenesis of atherosclerosis. Therefore, reducing LDL cholesterol (LDL-C) to the goals recommended by clinical practice guidelines, is essential to decrease the risk of vascular complications. Despite this, current LDL-C control is scarce, even in subjects with high and very high risk. This is mainly due to an insufficient intensification of lipid-lowering treatment. In this context, it is essential to prescribe the appropriate therapy, adjusted to patient’s needs based on their LDL-C and their vascular risk. Rosuvastatin, alone or in combination with ezetimibe, provides intensive LDL-C reductions (up to 50–55% and 60–75%, respectively), with a low risk of side effects and in an efficient manner, in patients both without and with established atherosclerotic vascular disease.

## 1. Introduction

Despite the continuous decrease in cardiovascular mortality over the last decades, vascular diseases remain the leading cause of death in Spain [1]. Thus, during the first semester of 2023, 27.1% of deaths were related to cardiovascular conditions, 25.8% to cancer and 11.5% to respiratory diseases [1]. The decrease in cardiovascular mortality has contributed significantly to the prolonging of the life expectancy of the Spanish population [1,2]. Thus, between 1980 and 2009, life expectancy increased by 6 years in Spain [2]. The decrease in vascular mortality contributed, by 63% in women and 53% in men, to this increase in longevity [2]. The factors that have motivated the decrease in coronary mortality in Spain have also been evaluated [3]. Between 1988 and 2005, coronary mortality decreased by 40%. Half of this decline was due to an improvement in the population’s cardiovascular risk factor profile, specifically, a reduction in cholesterol and blood pressure levels, a decrease in smoking and an increase in physical activity. However, there were opposing factors that increased its incidence, such as obesity and diabetes, and these negatively impacted on mortality. The other half of the decline was explained by the use of drugs in primary and, mainly, secondary prevention, and by better management during the acute coronary episode. These data are similar to those found in other countries, where, similarly, half of the benefits derived from the population’s improvement in cardiovascular risk factors and the other half from treatments used in the management of risk factors and cardiovascular disease [4]. In addition, a comprehensive approach is required in order to actually reduce cardiovascular disease. Thus, obesity is a recognized risk factor for the development of comorbid conditions such as cardiovascular disease and hypercholesterolemia. Achieving weight loss through a healthy lifestyle seems to be an ideal solution [5,6]. In addition, emerging risk factors, including sex-related factors (i.e., early menopause, gynecological tumors, etc.), should also be considered [7,8]. Given all the above, it seems evident that an optimal management of risk factors may have implications for the incidence of cardiovascular diseases and related mortality.

Hypercholesterolemia is not only a major, independent cardiovascular risk factor, but is also the underlying cause of atherosclerosis, particularly cholesterol that binds to low-density lipoproteins (LDL-C) and to apolipoprotein B-containing lipoproteins [9,10]. Clinical trials have shown that reducing LDL-C levels with lipid-lowering therapy is associated with a decrease in the risk of developing cardiovascular disease; the greater the reduction in LDL-C levels, the greater the protective effect against atherosclerotic cardiovascular disease [11]. In fact, there is no limit to the amount that LD-CL can be lowered while still reducing CV risk, and without a harmful effect [12]. Despite this, the control of hypercholesterolemia has been found to be suboptimal by all the studies that have evaluated this issue. In a review of 21 studies conducted between 2010 and 2014 that analyzed the rate of goal-attainment in patients in secondary prevention, the overall proportion of adequate control was only 31% [13].

As new drugs capable of reducing LDL-C concentrations to very low levels have become available, our knowledge of the relationship between cholesterol and vascular disease has improved and has motivated the downward modification of treatment goals. Those currently in force are the ones recommended by the European Society of Atherosclerosis (EAS) and the European Society of Cardiology (ESC) in their 2019 document [14] and by the European Society of Cardiology in their 2021 document [15]. These guidelines recommend the rapid attainment of LDL-C goals to maximize the preventive benefits [14,15].

The basis of the lipid-lowering treatment is statins. However, not all statins have demonstrated the same ability to reduce LDL-C, with atorvastatin and rosuvastatin having been found to be the most powerful statins for reducing it [14]. This review will focus on the importance of intensive rosuvastatin-based lipid-lowering therapy, researching its place in clinical practice.

## 2. Current Situation of Lipid Control

### 2.1. Europe

**Figure 1:**
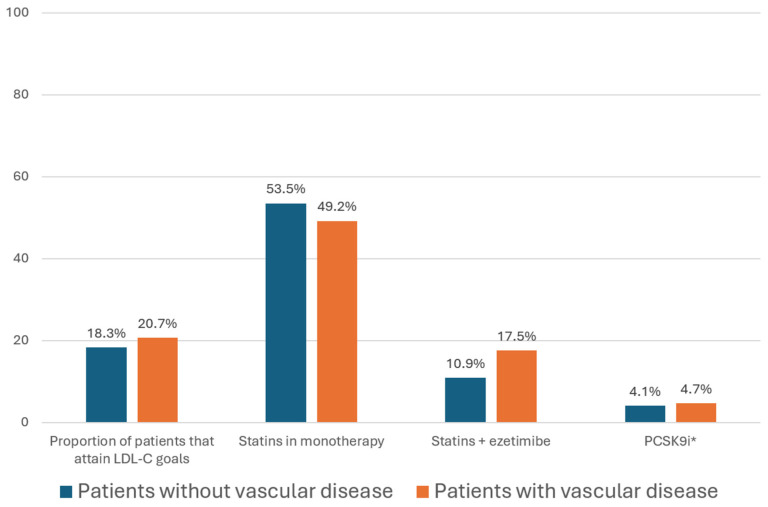
Proportion of patients that achieve LDL-C targets and proportion of patients using lipid-lowering drugs in the SANTORINI study according to the presence of vascular disease. LDL-C: low-density lipoprotein cholesterol; PCSK9i: proprotein convertase subtilisin/kexin type 9 inhibitors. Figure created with data from reference [16].

The SANTORINI study included more than 9000 patients at high and very high vascular risk from 14 European countries, including 990 subjects from Spain [16]. Overall, the percentage of patients who achieved LDL-C goals was 20.1%, with 24% among high-risk and 18.6% among very high-risk patients. The use of lipid-lowering drugs was suboptimal, with only 4.5% using proprotein convertase subtilisin/kexin type 9 inhibitors (PCSK9i), 24% combination therapy, and 21.8% not receiving any treatment. The mean LDL-C concentration in high-risk patients was 93 mg/dL, and in very high-risk patients it was 78 mg/dL (Figure 1) [16]. If these patients had achieved the recommended goals (<70 mg/dL and <55 mg/dL for patients at high and very high vascular risk, respectively), they would have decreased their LDL-C by an additional 23 mg/dL and would have avoided potential increases in vascular risk of between 12 and 15%. Unfortunately, these low rates of achievement of LDL-C deprived these patients of an additional benefit.

### 2.2. Spain

The dyslipidemia observatory was a study carried out exclusively in Spain by 435 physicians [17]. This study recruited 4010 patients from different clinical settings (34% from cardiology, 28% from primary care, 24% from internal medicine, and 14% from endocrinology). Overall, 31% had LDL-C levels within targets: 47%, 36%, 22% and 25% among low, moderate, high, and very high-risk patients, respectively. As in the SANTORINI study, the use of lipid-lowering therapies was suboptimal. Among the high and very high-risk participants, 7% and 1% did not receive any lipid-lowering treatment, 21% and 8% received low or moderate intensity statins, 44% and 38% received high intensity statins in monotherapy, 21% and 45% a combination of statins and ezetimibe, and 4% and 6% PCSK9i, respectively.

The SNAPSHOT study [18] included 443 patients with hypertension and dyslipidemia, with the aim of knowing how many of them were adequately controlled. The data were just as disappointing as in previous studies, with only 24% achieving the recommended targets, 16.7% among those at low or moderate risk, 21.8% in those at high risk, and 25.1% among those at very high risk. Furthermore, joint control of dyslipidemia and hypertension was only observed in 9% of the study population.

The low rate of goal achievement observed in previous studies represents a loss of opportunity for our patients. There is abundant evidence showing that the lower the cholesterol, the lower the rate of cardiovascular complications, without any threshold below which benefit is no longer observed and without an increase in the number of adverse effects [19]. Most studies on the benefit of treating hypercholesterolemia, however, have not focused specifically on the utility of achieving therapeutic goals. In the Treat Stroke to Target study [20], patients with a history of stroke were randomized to groups with an LDL-C goal of less than 70 mg/dL, the recommended goal for patients with vascular disease on the date the study began, or a less strict objective, aimed at maintaining LDL-C between 90 and 110 mg/dL. The arm that achieved the more strict objectives reduced the primary end point of ischemic stroke, acute myocardial infarction, new urgent coronary or carotid revascularization, or cardiovascular death by 22%, compared to the control group.

Remarkably, in all these studies, physicians erroneously perceived that their patients were better controlled than they were. Thus, in an epidemiological study carried out in Spain that included 300 primary care physicians [21], respondents considered that 61.5% of their patients were within LDL-C targets. This irrational optimism is perhaps also responsible for the systematic underestimation of risk. Although most of the patients who were recruited in the previous studies had a very high calculated vascular risk, the physician who enrolled them perceived their risk to be simply high. This fact had clinical relevance because underestimating the risk led to a less intensive search for objectives and contributed to obtaining fewer patients reaching the goal.

The results of these studies also showed that the use of combination lipid-lowering therapy was associated with a higher rate of goal achievement. In the SANTORINI study [16], 32% of patients receiving combination therapy were on target, compared to only 20.9% of patients receiving monotherapy treatment. Of note, combination treatment is associated with a greater decrease in cholesterol levels, as well as in the rate of cardiovascular complications [22]. This fact is even more relevant if the combination treatment is received in a single pill. In a retrospective analysis [23], it was observed that patients receiving combination treatment with statins and ezetimibe reduced their cholesterol levels more if both drugs were combined in a single tablet than if they were taken separately.

## 3. Role of Rosuvastatin in the Control of Cholesterol and the Reduction of Vascular Events

### 3.1. Efficacy

Rosuvastatin is a relatively hydrophilic, potent and highly selective statin for the enzyme 3-hydroxy-3-methylglutaryl-CoA reductase which significantly reduces cholesterol synthesis. Rosuvastatin undergoes limited metabolism, as approximately only 10% is metabolized by cytochrome P450, which translates into a low risk of interactions with other drugs. About 90% of the rosuvastatin is excreted unchanged in the feces and the remaining 10% in the urine. Age has no relevant impact on the pharmacokinetics of rosuvastatin, nor does kidney disease, except in the case of severe renal insufficiency, which markedly increases exposure to rosuvastatin [24,25].

Several clinical trials have analyzed the effects of rosuvastatin on LDL-C. In particular, the STELLAR study showed that it was the most powerful statin, since, after 6 weeks of treatment, rosuvastatin 10–40 mg was able to reduce LDL-C by 46–55%, compared to 37–51% with atorvastatin 10–80 mg, 28–39% with simvastatin 10–40 mg, and 20–30% with pravastatin 10–40 mg. Likewise, rosuvastatin increased HDL cholesterol by 8–10% (vs. 2–6%, 5% and 3–6%, respectively), and decreased triglyceride levels by 20–26% (vs. 20–28%, 12–15% and 8–13%, respectively) [26]. These results were confirmed in the VOYAGER study, a pooled analysis based on data from 32,258 individual patients of studies comparing the efficacy of rosuvastatin with that of atorvastatin or simvastatin [27]. A meta-analysis of 50 studies, with a total of 51,956 patients, analyzed the effectiveness of different statins in reducing LDL-C levels, with rosuvastatin being found the most potent statin [28]. This superiority has been confirmed in a more recent meta-analysis [29]. Nonetheless, rosuvastatin dosage reductions have been recommended in Asian patients, as it has been observed in pharmacokinetic studies that there is a two-fold increase in median exposure to rosuvastatin in Asian subjects when compared to Caucasian individuals [30,31]. On the other hand, the incidence of side effects seems lower with rosuvastatin than with atorvastatin [32], a finding also reached in real-life studies [33]. Likewise, rosuvastatin has not only demonstrated direct positive effects on the lipid profile, but could have additional benefits, due to its anti-inflammatory, antioxidant, antithrombotic and vascular protective properties, among other aspects [34,35,36,37,38].

**Figure 2:**
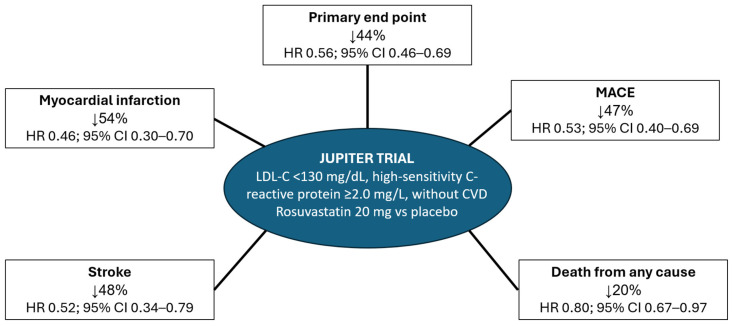
Effect of rosuvastatin vs. placebo on main clinical outcomes in the JUPITER trial. Primary endpoint: myocardial infarction, stroke, arterial revascularization, hospitalization for unstable angina or cardiovascular death. MACE: cardiovascular death, myocardial infarction, or stroke. CVD: cardiovascular disease. HR: Hazard Ratio. 95% CI: 95% confidence interval. LDL-C: low-density lipoprotein cholesterol. Figure made with data from reference [39].

The role of rosuvastatin in the prevention of vascular complications across the entire spectrum of patients with hypercholesterolemia has been evaluated in several studies. One of the most important was the JUPITER clinical trial [39], a phase III study, which included 17,802 subjects with LDL-C < 130 mg/dL, high-sensitivity C-reactive protein ≥ 2.0 mg/L, and without known cardiovascular disease. That is, these were patients without indications for treatment with statins at the time the study was carried out. Patients were randomized to receive rosuvastatin 20 mg or placebo. The study was stopped prematurely, after a follow-up of only 1.9 years, due to the beneficial effects of rosuvastatin in reducing events. LDL-C reductions with rosuvastatin were around 50%, in line with previous studies. Additionally, there was a 37% decrease in high-sensitivity C-reactive protein levels during the study, confirming the anti-inflammatory properties of rosuvastatin. Compared to placebo, rosuvastatin 20 mg significantly reduced the risk of the primary composite endpoint (myocardial infarction, stroke, arterial revascularization, hospitalization for unstable angina, and cardiovascular death) by 44% (projected 5-year NNT of 25) [40], as well as that of MACE by 47%. Of note, the JUPITER study is one of the few clinical trials with lipid-lowering treatments that has demonstrated a significant reduction in mortality from any cause (Figure 2) [39]. The results of the JUPITER study were consistent in the different subgroups of patients studied, and were independent of age, sex, the patient’s baseline cardiovascular risk, and the presence of diabetes or chronic kidney disease, emphasizing the benefits of rosuvastatin across the spectrum of patients with dyslipidemia [41,42,43,44,45,46]. Similarly, the HOPE-3 study found, in 12,705 patients without previous cardiovascular disease but with an intermediate cardiovascular risk, that after a median follow-up of 5.6 years, compared to placebo, the addition of rosuvastatin 10 mg to the treatment was associated with a significant reduction of 24% (HR 0.76; 95% CI 0.64–0.91; *p* = 0.002) in the risk of MACE (cardiovascular death, myocardial infarction or stroke); of 25% in the composite secondary variable of cardiovascular death, myocardial infarction, stroke, revascularization, heart failure or recovered cardiac arrest (HR 0.75; 95% CI 0.64–0.88; *p* < 0.001); 35% in the risk of myocardial infarction (HR 0.65; 95% CI 0.44–0.94); 30% in the risk of stroke (HR 0.70; 95% CI 0.52–0.95) and 32% in the need for revascularization (HR 0.68; 95% CI 0.48–0.95). Furthermore, the results were consistent within the different subgroups analyzed, including baseline cardiovascular risk, as well as lipid levels, C-reactive protein and blood pressure [47].

The role of rosuvastatin has been analyzed not only in patients in primary prevention, but different clinical trials and real-life studies have also been developed in subjects with established atherosclerotic vascular disease [25]. Thus, different clinical trials have shown that rosuvastatin improves the lipid profile (reduction of LDL-C and increase of HDL cholesterol) in this population, even to a greater extent than other statins, including atorvastatin in some of them [48,49,50,51,52,53,54,55,56]. Likewise, it has also been observed that treatment with rosuvastatin can reduce the volume of and stabilize atherosclerotic plaque, in addition to reducing the progression of carotid intima-media thickness and inflammation, as well as ventricular remodeling and myocardial fibrosis [49,50,51,52,53,55,57,58,59]. These positive results translate into a lower risk of recurrence of vascular events, as has been shown in different studies. Thus, in a recent real-life study of patients who had had an acute coronary syndrome, after one year of follow-up, the number of MACE, as well as its separate components, was found to be low and similar between patients treated with rosuvastatin and atorvastatin [60]. A similar trend has been observed in the LODESTAR clinical trial, in 4400 subjects with coronary artery disease who were followed for 3 years [61]. These results have also been observed in studies specifically performed in the Spanish population. Thus, in a nationwide retrospective and multicenter study, after a mean follow-up of 3 years, the recurrence rate of atherosclerotic vascular events was 2.73 cases/100 person-years for patients treated with atorvastatin vs. 2.34 cases/100 person-years for those treated with rosuvastatin, with no statistically significant differences between the two groups [62]. In the SAFEHEART study, which analyzed 1939 patients with familial hypercholesterolemia followed prospectively in Spain, after a median follow-up of 6.6 years, the incidence of atherosclerotic vascular events was similar between patients treated with atorvastatin and rosuvastatin (1.1 vs. 1.2 events/100 patient-years, respectively; *p* = 0.58) [63]. In summary, rosuvastatin has also been shown to be an effective and safe statin in patients with established atherosclerotic vascular disease [64].

### 3.2. Safety/Interactions

Clinical trials have shown that rosuvastatin is a very safe statin, with a very low risk of adverse events and drug interactions. Thus, for example, in the JUPITER study, rosuvastatin did not increase the risk of myopathy or cancer, and although in the HOPE-3 study there were more cases of muscle weakness or pain with rosuvastatin, the rates of discontinuations due to muscle symptoms, cases of rhabdomyolysis and myopathy were similar to those of the placebo group.

In the JUPITER study there was an increased risk of physician-reported diabetes, which was not the case in the HOPE-3 study. Additionally, in the JUPITER study, HbA1c increased from 5.7% to 5.9% with rosuvastatin (vs. 5.8% with placebo). Consequently, although the risk of diabetes was statistically increased with rosuvastatin in the JUPITER study, considering the robust reduction in vascular events, overall, it could be concluded that this increase was of little clinical relevance [39,47,65]. On the other hand, despite the fact that an increased incidence of proteinuria with high doses or rosuvastatin, particularly 40 mg/day, has been reported, this is a rare complication, with a good kidney prognosis, and normalizing observed after withdrawal of rosuvastatin [66].

Finally, since many patients with dyslipidemia have other associated cardiovascular risk factors and comorbidities, they are frequently polymedicated, which increases the risk of drug–drug interactions and potential side effects. In this context, unlike what happens with other statins such as atorvastatin, simvastatin and lovastatin, which are metabolized by the 3A4 isoform of cytochrome P450 (CYP3A4), rosuvastatin differs in its metabolization, which greatly reduces the risk of pharmacological interactions, and therefore confers greater safety, especially in polymedicated patients. This is important because numerous frequently used drugs such as verapamil, diltiazem, nifedipine, midazolam, alprazolam, erythromycin, fluoxetine, sertraline, amiodarone, mirtazapine, esomeprazole, omeprazole, digoxin and warfarin, among others, are metabolized through this route. Nonetheless, since levels of rosuvastatin markedly increase with the concomitant use of ciclosporin, co-administration of both drugs is contraindicated. Similarly, concomitant protease inhibitor use is not recommended due to the increase in rosuvastatin exposure with concomitant treatment [24,25].

### 3.3. Cost-Effectiveness

It is not only important to reduce LDL-C levels and vascular complications, but also to be efficient in the use of these therapies, that is, to ensure the greatest benefit with the lowest cost. In this sense, both rosuvastatin monotherapy and the combination of rosuvastatin and ezetimibe have been shown to be dominant treatments (more effective and less expensive) in a high proportion of patients, especially in those with a higher vascular risk (Table 1) [67,68]. Therefore, rosuvastatin is a high-intensity statin, which may contribute to the sustainability of the health care system [69].

## 4. Role of Rosuvastatin-Based Combination Lipid-Lowering Therapy

The IMPROVE-IT study demonstrated in subjects hospitalized for acute coronary syndrome that the combination of a statin with ezetimibe was more effective than statin monotherapy, not only in reducing LDL-C, but also in reducing recurrences of atherosclerotic vascular events, without increasing the risk of adverse effects [70]. Furthermore, the addition of ezetimibe to statins is much more effective than doubling the dose of statins in reducing LDL-C levels. This is not surprising, since statins and ezetimibe have complementary mechanisms of action (reduction of cholesterol synthesis and reduction of cholesterol absorption, respectively) that enhance their lipid-lowering action [24,25,71,72]. Thus, while potent statins at maximum doses achieve approximately an average reduction in LDL-C of around 50–55%, the decrease in LDL-C with the combination of potent statins and ezetimibe reaches approximately 60–75% [73].

Different clinical trials have specifically been developed to analyze the effects of the combination of rosuvastatin and ezetimibe on LDL-C levels and the achievement of lipid control objectives compared to rosuvastatin in monotherapy or other combinations of statins with ezetimibe in patients with hypercholesterolemia, mainly in subjects with a high vascular risk. In all of them, greater reductions in LDL-C and a greater degree of achievement of the objectives set by the clinical practice guidelines have been observed with the combination of rosuvastatin and ezetimibe, without increasing the risk of side effects (Table 2) [74,75,76,77,78,79,80,81].

Consequently, in those patients who require LDL-C reductions above 50%, the combination of rosuvastatin and ezetimibe offers additional reductions in LDL-C and a higher control rate [25,82,83,84].

## 5. Discussion

Hypercholesterolemia is directly involved in the etiopathogenesis of atherosclerosis [14]. Despite this, the percentage of patients whose LDL-C levels are within therapeutic targets is very low, including patients with a higher vascular risk [16,17,18]. Although several reasons have been proposed, it seems that underestimation of the risk, as well as an inadequate perception of actual LDL-C control, play essential roles, since they lead to insufficient intensification of lipid-lowering treatment, which is key to the improvement of these figures [17,21,85].

In this context, rosuvastatin, a high intensity statin that provides marked reductions in LDL-C levels, significantly reduces the risk of cardiovascular events and death, as the JUPITER trial demonstrated [39], in addition to its efficacy in secondary prevention patients, although evidence in these patients is less strong [48,49,50,51,52,53,54,55,56]. On the other hand, despite the fact that an increased risk of diabetes has been observed among predisposed patients, this is not clinically relevant, due to the cardiovascular benefits of rosuvastatin; particular attention should be paid, however, with these patients [39,65]. Moreover, the addition of ezetimibe may be helpful in this context, as lower doses of rosuvastatin may be required to attain LDL-C, thus, reducing the risk of developing diabetes [25,65].

From a clinical point of view, the step-by-step lipid-lowering treatment approach recommended by the European guidelines (starting with statins up to the maximum tolerated doses, if the objectives are not achieved, adding ezetimibe, and if this is not sufficient, adding PCSK9i) facilitates therapeutic inertia and delays goal achievement [14]. Different scientific societies, such as the Spanish Society of Cardiology and the Spanish Society of Arteriosclerosis, have advocated establishing an individualized lipid-lowering treatment strategy from the beginning, one aimed at achieving the required LDL-C target without delay. That is, given that the average reduction in LDL-C produced by each lipid-lowering therapy, alone or in combination, is already known, as well as the LDL-C levels and the LDL-C target required in each patient, it is possible to estimate the precise treatment for each subject (i.e., statins in monotherapy, combination of statins and ezetimibe, etc.) [73]. The application of this initial individualized approach can improve the degree of LDL-C control, even in patients at high/very high vascular risk [86]. In this context, rosuvastatin is a high-intensity statin which produces average reductions in LDL-C of around 50–55%, and when combined with ezetimibe, reductions of up to 60–75% [73], which undoubtedly contributes to the improvement of LDL-C control in our patients. In the light of evidence, and with the aim of “the earlier the better”, guidelines should clarify which patients would benefit more from monotherapy or combined therapy, particularly those patients at higher risk. In addition, future research is needed to clarify whether this approach will translate into a reduction of cardiovascular events compared to the traditional approach, as well as the role of rosuvastatin in this setting.

## 6. Conclusions

Reducing LDL-C to the recommended targets is essential for decreasing the risk of vascular complications. Unfortunately, the current control figures are very poor. To improve LDL-C control, especially in high/very high-risk patients, it is essential to prescribe the appropriate lipid-lowering treatment, adjusting to the specific needs of each patient. In these patients, rosuvastatin, alone or combined with ezetimibe, provides intensive reductions in LDL-C, with a low risk of side effects and a lower cost, which is associated with a lower risk of vascular complications, both in patients without previous vascular disease, and in those with established atherosclerotic vascular disease.

## Figures and Tables

**Table 1:** Estimated LDL cholesterol reduction of rosuvastatin and the rosuvastatin/ezetimibe combination, compared to atorvastatin, as well as the associated costs per cycle.

Treatment	LDL-C Reduction	Cost per Cycle (2018)	Treatment	LDL-C Reduction	Cost per Cycle (2021)
Rosuvastatin 10 mg	46%	87.73€	Rosu/EZE 10/10 mg	56.7%	340.5€
Atorvastatin 40 mg	49%	150.78€	Ator/EZE 40/10 mg	58.8%	396.03€
Rosuvastatin 20 mg	50%	175.46€	Rosu/EZE 20/10 mg	59.8%	422.23€
Atorvastatin 80 mg	50%	306.24€	Ator/EZE 80/10 mg	59.9%	530.94€

Ator: atorvastatin; EZE: ezetimibe; Rosu: rosuvastatin. Table created with data from references [67,68].

**Table 2:** Effect of the rosuvastatin/ezetimibe combination on LDL cholesterol in phase 3 clinical trials.

Study (Year of Publication)	Treatments	LDL-C Reduction (%)	Proportion of Patients That Attain LDL-C Targets (%)
EXPLORER (2007) [74]	RSV/EZ 40 mg/10mg	−70.0 (*p* < 0.001)	94.0 (*p* < 0.001)
	RSV 40 mg	−57.0	79.1
GRAVITY (2014) [76]	RSV/EZE 10 mg/10 mg	−59.7 (*p* < 0.05 vs. SIM/EZE 40/10 mg)	93.3 (*p* < 0.05 vs. SIM/EZE 40/10 mg)
	RSV/EZE 20 mg/10 mg	−63.5 (*p* < 0.05 vs. SIM/EZE 40–80/10 mg)	95.6 (*p* < 0.05 vs. SIM/EZE 40–80/10 mg)
	SIM/EZE 40 mg/10 mg	−55.2	87.4
	SIM/EZE 80 mg/10 mg	−57.4	88.6
MRS-ROZE (2016) [77]	RSV/EZE 5–20 mg/10 mg	−59.1 (*p* < 0.001)	94.1 (*p* ≤ 0.01)
	RSV 5–20 mg	−49.4	86.3
ROSE (2017) [78]	RSV/EZE 5–20 mg/10 mg	−59.5 (*p* < 0.001)	90.7 (*p* ≤ 0.01)
	RSV 5–20 mg	−51.1	72.9
SP-RE-003 (2018) [79]	RSV/EZE 5–20 mg/10 mg	−56.5 (*p* ≤ 0.01)	94.2 (*p* < 0.05)
	RSV 5–20 mg	−45.2	86.6
I-ROSETTE (2018) [80]	RSV/EZE 5–20 mg/10 mg	−57.0 (*p* < 0.001)	92.3 (*p* < 0.001)
	RSV 5–20 mg	−44.4	79.9

LDL-C: Low-density lipoprotein cholesterol; EZE: ezetimibe; RSV: rosuvastatin; SIM: simvastatin. Table created with data from references [74,76,77,78,79,80].
